# Heart Disease, Now What? Improving Quality of Life through Education

**DOI:** 10.3390/ijerph18063077

**Published:** 2021-03-17

**Authors:** Lisa Gomes, Cristina Liébana-Presa, Beatriz Araújo, Fátima Marques, Elena Fernández-Martínez

**Affiliations:** 1 Nursing School, Minho University, 4710-057 Braga, Portugal; lgomes@ese.uminho.pt; 2 SALBIS Research Group, Faculty of Health Sciences, Campus of Ponferrada, Universidad de León, 24401 Ponferrada, Spain; 3 Institute of Health Sciences, Universidade Católica Portuguesa, 4169-005 Porto, Portugal; baraujo@porto.ucp.pt; 4 Intensive Care Coronary Unit, Centro Hospitalar De Trás-Os-Montes E Alto Douro, E.P.E., 5000-508 Vila Real, Portugal; fatimaecmarques@sapo.pt; 5 SALBIS Research Group, Faculty of Health Sciences, Universidad de León, 24071 León, Spain; elena.fernandez@unileon.es

**Keywords:** coronary disease, cardiac rehabilitation, health education, quality of life, self-care

## Abstract

Introduction: The management of chronic illness assumes a level of demand for permanent care and reaches a priority dimension in the health context. Given the importance of nursing care to post-acute coronary syndrome patients, the objective of this study is to evaluate the impact of an educational intervention program on quality of life in patients after acute coronary syndrome. Method: Quasi-experimental study with two groups: an experimental group exposed to the educational intervention program and the control group without exposure to the educational intervention program. Results: The results showed statistically significant differences between both groups (*p* < 0.001). Although only valid for the specific group of subjects studied, the educational intervention program enabled significant gains in quality of life. Conclusions: According to the findings of the study, a systematized and structured educational program, integrated into the care organization and based on transition processes, is effective in developing self-care skills and improves the quality of life in patients after acute coronary syndrome.

## 1. Introduction

Cardiovascular diseases (CVD) are the main cause of death [1,2] decade after decade and self-care is not at the top of the pyramid of best practices for chronic disease management.

Changes in the population’s health/disease patterns pressure worldwide health care systems’ sustainability. These changes include an aging population, an increasing number of individuals with CVD risk factors, and the prevalence of multimorbidity [3]. The burden of heart disease in Europe indicates that we are presently far from achieving the expected success [4]. Health care systems believe that people who seek and need health care will obey the recommendations proposed by professionals.

Patients with heart disease have needs in various dimensions of their lives that require continuous health care, which the current systems have difficulties in satisfying. In the health–disease transition, rehabilitation depends largely on health literacy, adherence to rehabilitation programs, and the patient’s active participation in the management of their therapeutic regime [5]. Skills and ability development, behavior, and lifestyle changing is a challenge given the complex treatment regimes.

In the specific case of acute coronary syndrome (ACS), disease control requires a rigorous and long-lasting therapeutic plan. There is a need for patient and family involvement and compliance with the set of proposed recommendations [6]. Non-compliance with therapeutic adherence reaches such proportions that it can be considered a new cardiovascular risk factor [7].

Therefore, it is essential to develop strategies that promote greater adherence to the prescribed treatment. These educational programs can represent an important strategy in the fight against heart disease because they enable the development of self-care skills necessary for patients to reach their potential and quality of life (QoL) [8].

However, cardiac patients have difficulties in identifying and managing signs and symptoms related to heart disease, in adhering to therapy, in performing daily life activities, and even in interpersonal management [9]. For these reasons, self-care education is fundamental because it is about influencing behavior change by increasing knowledge, changing attitudes, and developing skills. Education goals include cardiac patients’ participation in decision making for lifelong continuing care, awareness-raising, and functional performance [10].

The educational process is gradual, systematized, and personalized. In clinical practice, nurses find it difficult to implement these educational programs due to the high cost of hospitalizations and scientific advances in treatment that have shortened the length of hospital stays for patients with heart disease. This implies that in the rehabilitation plan the educational process begins on the first day of hospitalization [11].

Cardiac rehabilitation programs (CRP) have a strong educational component. Recognized in recent decades as an intervention with a cost-effective impact, CRP assume an integral part of the multidimensional treatment. They improve prognosis, reduce the number of readmissions and health expenses, as well as increase QoL [12]. However, in Portugal, as in other countries, the rate of admission to cardiac rehabilitation programs is very low [13]. The uncertainty about what will happen after discharge and how the patient can adapt to their new health condition justifies a care practice that facilitates the process of making informed decisions. Given that not everyone has access to CRP, the need for implementing educational interventions during hospitalization becomes even more important to improve patient outcomes.

Aware of the importance of nursing care for the post-ACS patient, the objective of this research is to design, implement, and evaluate the impact of an educational intervention program during the hospitalization period on the development of self-care skills and QoL. This article also adds data to the study previously published by Gomes and Reis (2019), and contributes to a better understanding of the importance of nurse-led educational intervention [14].

## 2. Materials and Methods

### 2.1. Study Design and Setting and Procedure

The authors conducted quasi-experimental research, with the establishment of two groups—an experimental group, and a control group. The control group received standardized nursing care, and the experimental group, in addition to the standardized nursing care, had access to the educational intervention program. The 67 participants were adult/elderly patients diagnosed with ACS, hospitalized at an intensive care coronary unit (ICCU) in the northern region of Portugal, who spoke and understood Portuguese, had preserved cognitive and verbal ability, and agreed to participate in the study [14].

However, we encountered the impossibility of randomizing the groups due to the organization of the ICCU and the cardiology unit. If randomization were the choice, the participants of the control group would attend the educational intervention program since the ICCU is an open space and the cardiology unit does not have single rooms. To solve this problem, we opted for the consecutive series method, which consisted of testing the educational intervention in a non-parallel way [15]. Throughout 2016, during two-week periods, patients were included in the intervention group and, the following week new patients were included in the control group and so on, until reaching the required number of participants for our study. Despite the non-randomization, this method allows the same probability of the patient being included in one of the groups [16]. Table 1 shows the distribution of the groups by weeks/months.

Each phase of the program has specific objectives, as well as different strategies for action. Table 2 summarizes these phases. The four sessions provided moments for intrinsic motivation, learning, and reflection. Nurse–patient interviews conducted during hospitalization and one month after discharge was the strategy adopted for data collection. Due to the geographical dispersion of the hospital’s affluence area, authors opted to contact patients by telephone.

The ethics committee and administration council of the institution and the medical director of the ICCU approved the study protocol. This study did not entail any cost to the participant or institution. It did not involve therapeutic actions; invasive, radiological procedures; or the collection of biological products. Each patient filled out a declaration of informed consent.

### 2.2. Educational Intervention Program

**Figure 1:**
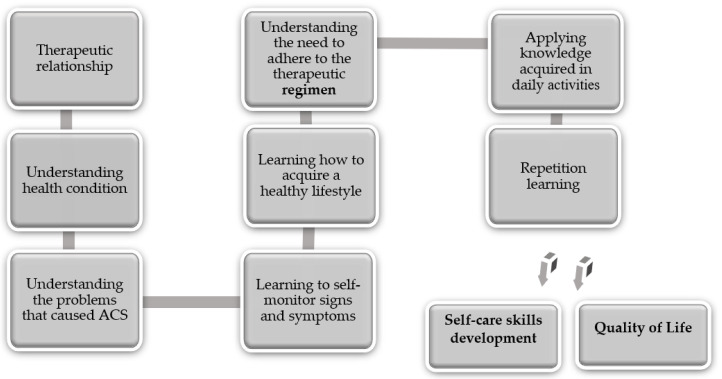
Educational intervention program framework and outcome indicators.

To obtain significant learning for the development of self-care skills, the program had a structured and systematic approach with four sessions [14,17] and involved three educational areas. At the end of the sessions, the patient should be able to: (i) know the risks of heart disease (cognitive area); (ii) know how to control the signs and symptoms of heart disease and know how to perform their most instrumental self-care (psychomotor area); (iii) understand that changing their behaviors increases their QoL (affective area). Figure 1 illustrates the educational intervention program framework and the outcome indicators.

Given that hospitalization is increasingly shorter and that there is a need to repeat information for processing, the authors used the theoretical model of social learning of Bandura [18]. Based on the statement of Bandura’s theory of self-efficacy, that expectations of self-efficacy are dependent on contexts and situations of achievement, patients are encouraged to imagine success, anticipate potential outcomes, and so respond confidently with more adaptive strategies to overcome barriers. The patients need to trust their abilities to achieve the proposed goals. The more confident and motivated the patient, the greater the success and opportunity to enhance a new health behavior.

The educational intervention program had the following pedagogical strategies: (i) interviews with the nurse rehabilitation specialist; (ii) an educational video; (iii) checklist/pamphlet; (iv) telephone follow-up interview. The first session began in the ICCU, 12 to 24 h after hospitalization and depending on the hemodynamic stability of the patient. The nurse rehabilitation specialist conducted an interview with didactic resources; the authors opted for the visualization of a video with the following themes and contents:

- How does the heart function?
- What is coronary heart disease?
- Diagnosis of coronary disease;
- Therapeutic management;
- Modifiable risk factors;
- Healthy lifestyle.

The second session was a group session and held the following day, aimed at identifying the patient’s knowledge regarding the contents exposed in the video, and clarifying doubts and misassumptions. The authors used this strategy since the patients’ beliefs about the disease and the personal experience also result from the social experience. Loring and Holman explain that social persuasion can improve the perception of self-efficacy [19]. During the third session held on the day of hospital discharge, the nurse used a checklist with the contents of the video. The checklist had the following purpose: (i) organize and systematize the information so that the nurse can identify difficulties, and (ii) serve as a leaflet for patient guidance. The fourth and last session occurred one month after discharge, with the aim of monitoring the patient and reinforcing information.

### 2.3. Instruments Used to Collect Data

For data collection, authors used a sociodemographic and clinical context questionnaire, the therapeutic self-care scale and the MacNew heart disease health-related QoL questionnaire.

Doran et al. [20] developed the therapeutic self-care scale (TSCS) with the objective of assessing the capacity for self-care in acute care contexts. This scale assesses the person’s ability to perform four categories of self-care activities: taking medication as prescribed by the doctor; identifying and managing symptoms; performing activities of daily living; and managing changes in health status. The maximum score is 60 points and corresponds to a high level of performance in therapeutic self-care. The original version of the instrument by Doran et al. was translated, validated, and adapted for the Portuguese population by Cardoso, Queirós, Fontes Ribeiro, and Amaral [21].

The MacNew heart disease questionnaire is a self-administered modification of the original quality of life after myocardial infarction (QLMI) instrument [22]. It assesses the feelings of patients who have suffered an acute myocardial infarction and are attending a CRP. Höfer et al. [23] states that the MacNew QLMI scale is a specific QoL assessment instrument, but it is used also in other cardiac pathologies, such as angina. To calculate the scores of the questionnaires, the authors used the average of all items and for the total QoL. For presentation and interpretation of results, the variable QoL has the following intervals: 1 to 3, 3 to 5, and 5 to 7. Patients with averages in the range 1 to 3 have worse QoL, patients with averages between 3 and 5 are considered to have moderate QoL, and high QoL is assumed for patients with an average between 5 and 7 points. Leal et al. [24] validated the MacNew QoL version for the Portuguese population.

### 2.4. Data Analysis

For data treatment, the authors used descriptive and inferential statistical techniques and used the Statistical Package for Social Science (SPSS) software, version 23 for statistical analysis. The statistical techniques applied were frequency (absolute and relative), measures of central tendency (arithmetic mean and median), measures of dispersion or variability (minimum value, maximum value, and standard deviation), and tests (Chi-square test, Fisher’s exact test, Mann–Whitney U test, Wilcoxon test, Spearman’s correlation coefficient significance test, and the Shapiro–Wilk test as a normality test). For all tests, the value of 0.05 was set as the limit of significance, that is, the null hypothesis was rejected when the probability of type I error (probability of rejection of the null hypothesis when it was true) was lower than the set value, when *p* < 0.05, that is, *p* < 5%.

## 3. Results

The results obtained for sociodemographic characteristics are set out in Table 3. In both groups, the majority of the patients were male, with the percentages being 90.6% in the experimental group and 71.4% in the control group. The ages of the patients in the experimental group extended between 40 and 80 years, with an average age of 57.72 ± 10.55 years. In this group, 28.1% of patients were under 50 years old; the same percentage was between 50 and 60 years old and between 60 and 70 years old. Half of the patients are older than 56.50 years old and the frequency distribution departed significantly from the characteristics of a normal or Gaussian curve (*p* = 0.022). We found that most patients of both groups reported being married or cohabiting, with percentages of 81.3% and 60.0%, respectively, in the experimental group and in the control group. In the experimental group, 43.8% of the patients had primary-level education, and in the control group, this percentage was even higher at 74.3%. No participant was illiterate.

The results illustrated in Table 4 allow us to know the clinical characteristics of the patients. The authors found that most elements of both groups had acute myocardial infarction diagnosis, with the percentages being 90.6% and 88.6%, respectively in the experimental and control group. This was followed by unstable angina (9.4% and 11.4%). The Chi-square test revealed that the observed difference is not statistically significant (*p* = 0.784). As for risk factors, it appears that in both groups, dyslipidemia predominates, with percentages of 78.1% and 71.4%, followed by arterial hypertension, with percentages of 53.1% and 54.3%, followed by smoking—37.5% and 34.3%, and diabetes mellitus—34.4% and 31.4%, respectively. It appears that the most common risk factors are present in both groups. The Chi-square test revealed that the differences observed between the two groups are not statistically significant (*p* = 0.999). Regarding the weight status, 37.5% of the patients in the experimental group were revealed to be in the pre-obesity situation, followed by 34.4% classified as Stage I obese. In the control group, 45.7% of the patients are pre-obese and 22.9% classified as Stage I obese. Through the application of the Mann–Whitney U test, to compare the body mass index, authors found that the differences observed between the two groups were not statistically significant (*p* = 0.292). Above-normal weight predominates in both groups.

The results also suggest improvements in the development of self-care skills and QoL in patients undergoing an educational intervention. Applying the Wilcoxon test to compare the two groups between each evaluation moment, the authors verified the existence of statistically significant differences in both cases (*p* < 0.001). The comparison of the mean and median values suggests that, between the first and in the second evaluation, the elements of the experimental group tended to improve their therapeutic self-care, while in the elements of the control group the trend was the opposite, that is, there was a deterioration in therapeutic self-care (Table 5). Authors also observed that all patients of the experimental group obtained results equal to 60 points (maximum value of the therapeutic self-care assessment scale). Consequently, the average value was 60.00 ± 0.00 points, the median had the same value, and the frequency distribution cannot be considered normal (*p* = 0.000). In the control group, the results ranged between 21 and 58 points, with an average value of 38.77 ± 7.74 points. It appears that half of the patients in this group had values equal to or greater than 39 points and the frequency distribution reveals characteristics similar to that of a normal distribution (*p* = 0.883). The application of the Mann–Whitney U test revealed the existence of significant differences between the two groups (*p* < 0.001) and a comparison of the values of the measures of central tendency reveals that the participants in the experimental group indicated better therapeutic self-care than those in the control group.

The results illustrated in Table 6 regarding the MacNew questionnaire for the patients in the experimental group showed minimum values and maximum values between 3.22 and 6.26 points, with an average value of 4.74 ± 0.65 points. This means that patients on average perceived their QoL positively. Half of the elements in this group showed results equal to or greater than 4.76 points, and the frequency distribution departed significantly from the characteristics of a normal distribution (*p* = 0.114). In the control group, we observed results between 1.74 and 4.96 points. The average value was 3.78 ± 0.66 points, with a median of 3.74 points. The frequency distribution can be considered normal (*p* = 0.249).

For the physical dimension of QoL, the authors observed, in the experimental group, results that ranged between 3.20 and 5.60 points, with an average value of 4.34 ± 0.63 points. Half of the respondents in this group had values equal to or greater than 4.40 points, and the frequency distribution can be considered normal (*p* = 0.375). In the control group, the authors observed values between 2.40 and 5.00 points, with an average of 3.43 ± 0.59 points. Half of the elements in this group obtained results above 3.40 points, which shows that the perception of the QoL is close to the value of 3.50 points that represents the average value of the scale. The values are lower than those of the experimental group. The frequency distribution departed significantly from a normal distribution (*p* = 0.182).

In the emotional dimension for the experimental group, values between 3.21 and 6.57 points were observed, with a mean value of 4.80 ± 0.77 points and a median of 4.93 points. The frequency distribution showed characteristics close to a normal curve (*p* = 0.687). In the control group, the observed values were between 1.43 and 5.00 points, with an average of 3.84 ± 0.74 points. Half of the individuals that constituted this group obtained results above 3.93 points and the frequency distribution departed significantly from the characteristics of a normal curve (*p* = 0.029).

In the social-life dimension, the authors found in the experimental group values between 3.00 and 6.67 points, with the mean value being 5.29 ± 0.79 points. Half of the elements in this group showed results greater than 5.33 points. The frequency distribution cannot be considered normal (*p* = 0.042). For the control group, authors found values between 1.83 and 6.17 points, with an average of 4.17 ± 0.91 points. Half of the sample elements showed values equal to or greater than 4.17 points. The frequency distribution shows characteristics close to a normal curve (*p* = 0.543).

Applying the Mann–Whitney U test, the authors can conclude that between both groups there is a statistically significant difference (*p* < 0.001) in global, physical, emotional, and social QoL. The comparison of the values of the measures of central tendency reveals that the elements of the experimental group showed significantly better QoL than those of the control group.

## 4. Discussion

As for the sociodemographic characterization, the authors found that in both groups, the patients are mostly male, with low literacy. The average age of the participants in the experimental group is 57.72, while the average age of the patients in the control group is 65.09. These results reflect the profile of coronary patients in the Portuguese population, according to governmental reports [25], in which the prevalence of coronary disease is higher in males, with low literacy and incidence between 50 and 70 years old. Regarding the level of education, the findings of this study revealed that for individuals with a low level of education, risk factors were more prevalent. These results were consistent with the findings of the study conducted by Marques da Silva et al. (2019). They studied the prevalence of cardiovascular risk factors and other comorbidities in patients with hypertension in Portuguese primary health care populations. However, despite the higher prevalence of individuals with low levels of education, it is worth highlighting the trend of a gradual increase in risk factors among individuals with higher education levels [26,27].

There was also a higher prevalence in both groups of married/cohabiting patients. In the Okwose et al. [28] study, patients reported that family members are a strong motivating factor. Having a partner or relative to support them and participate in new activity routines is important. Family can promote patients’ emotional support and enable therapeutic regimen adherence.

Data suggests that the educational program promotes improvements in the cardiac patient’s self-care skills and in global, physical, emotional, and social QoL. The program made patients more proactive in decision-making and assuming responsibilities for their health conditions. In the Riegel et al. [29] study, the behavior-change factors address habits, motivation, decision-making, and the challenges of persistence. However, it is important to consider that illness-related factors address specific issues that make self-care exceedingly difficult—multimorbidity, symptoms, and stressful life events.

The results of the Goodman, et al. [30] study carried out in patients with decompensated heart failure (HF), suggest that low scores in self-care skills can be related to difficulties in decision-making and lack of motivation, and not so much to learning difficulties. Therefore, in the Rice et al. [31] study, results suggest that nurse-led patient education for adults with HF improves QoL and reduces hospital admissions and readmissions—a major cause of health-care costs [32]. However, for this study the authors did not considered readmissions as an outcome. The randomized controlled trial study conducted in Iran with 60 patients that had HF also concluded that a self-management education program is an appropriate strategy for improving QoL in patients with HF [33].

Educational programs implemented during hospitalization aim to enable patients to deal more effectively with the chronic disease after hospital discharge. Improving patient’s health outcomes by increasing knowledge and behaviors related to the cause of the disease, treatment, and coping strategies can prevent complications and better QoL [34]. In our opinion, this educational program can be adapted and implemented among other chronic disease patients, since teaching skills can be generalized.

The results indicate that behavioral changes and self-care skills depend on educational programs for patients to learn and practice. The educational sessions are not just for transmitting information but also should involve moments of elucidation, reflection, and discussion accompanied by didactic material such as pamphlets and audiovisual media for better understanding. For practice implications, Rice et al. [31] considered diverse pedagogical methods, such as nurse-led education strategies.

The study design should be considered as a limitation; it would have been interesting to compare two intervention groups with each other. Furthermore, it would be desirable to carry out longitudinal studies to confirm the differences between the groups in the long term after the intervention.

## 5. Conclusions

This study revealed a positive relationship between the development of self-care skills and the QoL of the post-ACS patient. However, the period of one month between the two assessment moments (hospitalization and one month after discharge) may not have been enough to observe permanent changes in self-care skills and QoL. In future studies, it will be necessary to evaluate the effectiveness of the educational intervention program in a longer follow-up period.

Knowledge alone does not influence self-care behaviors. It is a complex decision-making process. The patient uses values and experiences in decision-making and these experiences emerge from situational awareness, their perceptions, and the meanings given to the cardiac event. Each option and response create a set of standards.

In making complex decisions, the nurse supports and helps the post-ACS patient to recognize decision-making situations and provide strategies to facilitate effective responses.

The nurse’s educational intention alone is not enough. This intentionality involves implementing educational interventions guidelines, so that patients and families are informed (knowledge development), trained (skills development), and involved in their health care. Nurses being patients’ partners in decision-making can indicate the path towards adaptation, autonomy, and QoL, because these decisions will be compatible with personal life goals.

## Figures and Tables

**Table 1:** Distribution of participants by the experimental and control groups.

Week	March	April	May	June	July
1st Week	Experimental	Control	Experimental	Control	Experimental
2nd Week	Experimental	Control	Experimental	Control	Experimental
3rd Week	Control	Experimental	Control	Experimental	Control
4th Week	Control	Experimental	Control	Experimental	Control

**Table 2:** Sessions developed during the educational intervention program for patients with acute coronary syndrome.

Sessions	Education Strategies	Objective
1st session (1st day of hospitalization)	Video/nurse and patient session	Develop patient’s awareness of their chronic disease Describe heart function and disease Improve adherence to treatment Signs and symptoms management Medication management Promote lifestyle changes Develop healthy eating habits Practice physical activity and exercise Express and understand psychological aspects
2nd session	Group session	
3rd session (Hospital discharge day)	Patient and family session	
4th session (1 month after hospital discharge)	Telephone follow-up	

**Table 3:** Sociodemographic characteristics.

		Group				
Variables		Experimental n = 32 (100%)		Control n = 35 (100%)		*p* Value
Sex	Female	3 (9.4%)		10 (28.6%)		0.047
	Male	29 (90.6%)		25 (71.4%)		
Marital Status	Married/Cohabiting	26 (81.3%)		21 (60%)		0.036
Education Level	Primary (elementary) school	14 (43.8%)		26 (74.3%)		0.102
		M ± SD	Min–Max	M ± SD	Min–Max	0.015
Age		57.72 ± 10.55	40–80	65.09 ± 11.05	45–89	

Note: M; Mean. SD; Standard Deviation. Min–Max; Minimum–Maximum.

**Table 4:** Clinical characteristics of the groups.

		Group		
Variable		Experimental n = 32 (100%)	Control n = 35 (100%)	*p* Value
Medical diagnostic	Acute myocardial infarction	29 (90.6%)	31 (88.6%)	0.784
	Unstable chest angina	3 (9.4%)	4 (11.4%)	
Risk factors	Dyslipidemia	25 (78.1%)	25 (71.4%)	0.999
	Arterial hypertension	17 (53.1%)	19 (54.3%)	
	Smoking	12 (37.5%)	12 (34.3%)	
	Diabetes mellitus	11 (34.4%)	11 (31.4%)	
Weight status	Pre-obesity	12 (37.5%)	16 (45.7%)	0.292
	Stage I obesity	11 (34.4%)	8 (22.9%)	

**Table 5:** Pre-intervention and post-intervention therapeutic self-care assessment scale results.

		Pre-Intervention Group			Post-Intervention Group		
Questionnaire		Experimental n = 32 (100%)	Control n = 35 (100%)	*p*-Value	Experimental n = 32 (100%)	Control n = 35 (100%)	*p*-Value
Therapeutic self-care	M ± SD	58.31 ± 1.20	43.54 ± 6.57	<0.001	60 ± 0.00	38.77 ± 7.74	<0.001
	Me	58	43		60	39	
	Min–Max	54–60	31–60		60–60	21–58	
	*p*-value *	<0.001	0.299		0.00	0.883	

Note: M; Mean. SD; Standard Deviation. Me; Median; Min–Max; Minimum-Maximum. * *p*-values for normal distribution.

**Table 6:** Quality of life assessment scale results.

		Group		
Questionnaire QoL		Experimental n = 32 (100%)	Control n = 35 (100%)	*p*-Value
Quality of life—global	M ± SD	4.74 ± 0.65	3.78 ± 0.66	<0.001
	Me	4.76	3.74	
	Min–Max	3.22–6.26	1.74–4.96	
	*p*-value *	0.114	0.249	
Quality of life—physical	M ± SD	4.34 ± 0.63	3.43 ± 0.59	<0.001
	Me	4.40	3.40	
	Min–Max	3.20–5.60	2.40–5.00	
	*p*-value *	0.375	0.182	
Quality of life—emotional	M ± SD	4.80 ± 0.77	3.84 ± 0.74	<0.001
	Me	4.93	3.93	
	Min–Max	3.21–6.57	1.43–5.00	
	*p*-value *	0.687	0.029	
Quality of life—social	M ± SD	5.29 ± 0.79	4.17 ± 0.91	<0.001
	Me	5.33	4.17	
	Min–Max	3.00–6.67	1.83–6.17	
	*p*-value *	0.042	0.543	

Note: M; Mean. SD; Standard Deviation. Me; Median; Min–Max; Minimum-Maximum. * *p*-values for normal distribution.
